# AI in steatohepatitis diagnostics: precision beyond the microscope

**DOI:** 10.1097/MS9.0000000000003997

**Published:** 2025-09-30

**Authors:** Ayesha Ejaz, Mehreen Mushtaq Ahmed, Maliha Khalid, Muhammad Talha, Aminath Waafira

**Affiliations:** a Department of Medicine, King Edward Medical University, Lahore, Pakistan; b Department of Medicine, Hayatabad Medical Complex, Peshawar, Pakistan; c Department of Medicine, Jinnah Sindh Medical University, Karachi, Pakistan; d Department of Medicine, The Maldives National University, Malé, Maldives

**Keywords:** artificial intelligence, digital pathology, liver fibrosis, non-alcoholic steatohepatitis

## Abstract

Non-alcoholic steatohepatitis (NASH), the progressive form of nonalcoholic fatty liver disease (NAFLD), is a major global health burden without curative therapy. Conventional histological diagnosis is limited by interobserver variability and insensitivity to subtle changes, necessitating more objective diagnostic tools. Artificial intelligence (AI), integrated with digital pathology techniques such as second harmonic generation/two-photon excitation (SHG/TPE) fluorescence imaging, provides quantitative and reproducible assessment of steatosis, ballooning, and fibrosis. Recent studies have demonstrated strong correlations between AI-derived and pathologist-assigned grades, underscoring AI’s potential to enhance diagnostic precision and reproducibility. However, challenges including high operational costs, limited large-scale validation, population heterogeneity, and lack of regulatory frameworks remain barriers to clinical translation. Expanding research, developing standardized protocols, and establishing robust policies are essential for widespread adoption. AI-based pathology could revolutionize the diagnostic paradigm for liver disease, enabling earlier detection, improved patient stratification, and precision care in NASH.

*To the editor,*

Nonalcoholic steatohepatitis (NASH), a progressive form of non-alcoholic fatty liver disease (NAFLD), is one of the leading causes of chronic liver disease across the world^[1]^. NAFLD is the most common chronic liver disease, with its prevalence in 25% of the global population^[2]^. It has no recognized treatments despite being linked to a significant health and financial burden. Conventional histological analysis, which is considered the standard for diagnosis, has some major shortcomings. Semiquantitative grading methods are not very sensitive to change, and subjective interpretation of lesions led to significant rates of variability both among and between the observers^[1]^. The use of AI for pathological diagnosis is increasing day by day, with beneficial findings mainly for the tumorous disorders^[2]^.

AI has found its applications in various medical domains, which include radiological image diagnosis, clinical diagnosis, analysis of clinical records, genomic analysis, illness risk prediction, and development of new medications. An objective, precise, and quick AI-based diagnosis is greatly needed for steatohepatitis because interobserver variation is inevitable and inherent in human-based pathological diagnosis of NAFLD/NASH^[2]^. Digital pathology (DP) refers to the tools used to digitize pathology slides, where second harmonic generation/two-photon excitation (SHG/TPE) fluorescence is an example, which is a stain-free imaging approach allowing the analysis of fine structural details. AI models can quantitatively analyze the relative proportion of different morphological features, including those that are not part of the histological classifications^[1]^.

Over the recent years, multiple studies have demonstrated the effectiveness of AI in the pathological diagnostics of chronic liver diseases. Mizuoch *et al* trained AI with liver biopsies from individuals with chronic hepatitis and steatohepatitis, which was then tested on patients with liver diseases. AI’s measurement of steatosis was found to correlate strongly with that by histopathologists (*R* = 0.78, *P* < 0.001). Similar results were found for ballooning and fibrosis^[3]^. Another study in Thailand also found an association of 68% between the fibrosis grading by AI and pathologists, being highest when differentiating between advanced and non-advanced fibrosis (89%). This highlights the pivotal role AI may potentially play in the diagnostics of liver pathologies with further technological advancements^[4]^.

Despite the promising results, there are still many challenges to overcome before the universal application becomes practicable. One of the major hurdles is the high costs of operation. Moreover, for an AI-based tool to be implementable in the diagnostics of rare liver diseases, it must reflect the heterogeneity of the global population, rather than a focused subset. To add to that, the limited number of studies and the lack of large-scale trials on the matter limit the reliability and validity of the tools prior to considering their widespread application. Lastly, the lack of safety regulations issued by governing bodies poses an ethical dilemma in their extent of implementation^[5]^.

In conclusion, AI’s integration exhibits a notable advancement in the landscape of liver pathology. Policymakers should introduce funding schemes to aid widespread accessibility of the technology among the populace, and must collaboratively carry out further trials with researchers to ensure the validity of the diagnostic tools. Moreover, the development of strict policies by the respective authorities is necessary, allowing global incorporation of AI-based tools, which could revolutionize the realm of pathological diagnostics of liver diseases. This letter to editor is in compliance with the TITAN guideline^[6]^.

## Data Availability

No data were generated for this manuscript.

## Research registration unique identifying number (UIN)

Name of the registry: N/A; Unique Identifying number or registration ID: N/A; Hyperlink to your specific registration (must be publicly accessible and will be checked): N/A.
